# Gene-based single nucleotide polymorphism discovery in bovine muscle using next-generation transcriptomic sequencing

**DOI:** 10.1186/1471-2164-14-307

**Published:** 2013-05-07

**Authors:** Anis Djari, Diane Esquerré, Bernard Weiss, Frédéric Martins, Cédric Meersseman, Mekki Boussaha, Christophe Klopp, Dominique Rocha

**Affiliations:** 1 INRA, SIGENAE, UR 875, INRA Auzeville, BP 52627, 31326 Castanet-Tolosan Cedex, France; 2 INRA, UMR 444, Laboratoire de Génétique Cellulaire, INRA Auzeville, BP 52627, 31326 Castanet-Tolosan Cedex, France; 3 GeT-PlaGe, Genotoul, INRA Auzeville, BP 52627, 3132, Castanet-Tolosan Cedex, France; 4 INRA, UMR 1313 GABI, Unité Génétique Animale et Biologie Intégrative, Domaine de Vilvert, 78352 Jouy-en-Josas, France

**Keywords:** Single Nucleotide Polymorphism, Cattle, Muscle, RNA-Seq, Beef, Non synonymous coding variants

## Abstract

**Background:**

Genetic information based on molecular markers has increasingly being used in cattle breeding improvement programmes, as a mean to improve conventionally phenotypic selection. Advances in molecular genetics have led to the identification of several genetic markers associated with genes affecting economic traits. Until recently, the identification of the causative genetic variants involved in the phenotypes of interest has remained a difficult task. The advent of novel sequencing technologies now offers a new opportunity for the identification of such variants. Despite sequencing costs plummeting, sequencing whole-genomes or large targeted regions is still too expensive for most laboratories. A transcriptomic-based sequencing approach offers a cheaper alternative to identify a large number of polymorphisms and possibly to discover causative variants. In the present study, we performed a gene-based single nucleotide polymorphism (SNP) discovery analysis in bovine *Longissimus thoraci*, using RNA-Seq. To our knowledge, this represents the first study done in bovine muscle.

**Results:**

Messenger RNAs from *Longissimus thoraci* from three Limousin bull calves were subjected to high-throughput sequencing. Approximately 36–46 million paired-end reads were obtained per library. A total of 19,752 transcripts were identified and 34,376 different SNPs were detected. Fifty-five percent of the SNPs were found in coding regions and ~22% resulted in an amino acid change. Applying a very stringent SNP quality threshold, we detected 8,407 different high-confidence SNPs, 18% of which are non synonymous coding SNPs. To analyse the accuracy of RNA-Seq technology for SNP detection, 48 SNPs were selected for validation by genotyping. No discrepancies were observed when using the highest SNP probability threshold. To test the usefulness of the identified SNPs, the 48 selected SNPs were assessed by genotyping 93 bovine samples, representing mostly the nine major breeds used in France. Principal component analysis indicates a clear separation between the nine populations.

**Conclusions:**

The RNA-Seq data and the collection of newly discovered coding SNPs improve the genomic resources available for cattle, especially for beef breeds. The large amount of variation present in genes expressed in Limousin *Longissimus thoracis*, especially the large number of non synonymous coding SNPs, may prove useful to study the mechanisms underlying the genetic variability of meat quality traits.

## Background

Cattle (*Bos taurus*) are considered to have been one of the first animals domesticated by man for agricultural purposes. Approximately 10,000 years ago, cattle ancestors (aurochs) were tamed to provide milk, meat and hides and for draft purposes [1]. *Bos taurus* was also one of the first animal species to enter the genomics era. In the past few years, genetic information based on molecular markers has increasingly been used in cattle breeding improvement programmes, as a mean to improve conventionally phenotypic selection, particularly for traits with low heritability or for which measurement of phenotype is difficult, expensive, only possible late in life, sex-limited or not possible on selection candidates [2]. Advances in molecular genetics have led to the identification of several genes or genetic markers associated with genes that affect economic traits [3-10]. For example, the non conservative K232A substitution in the *acylCoA:diacylglycerol acyltransferase* (*DGAT1*) gene has a major effect on milk yield and composition [5]. Several of these genetic markers are now available and used in industry marker-assisted selection programmes [11,12].

Because of its economical importance *Bos taurus* was one of the first mammals to have its genome sequenced. In August 2006, the sequence of the cattle genome was released by the Human Genome Sequencing Center at Baylor College of Medicine [13]. During the sequencing more than 2.2 million putative single nucleotide polymorphisms (SNPs) were identified and deposited in public databases [14]. The Bovine Genome Sequencing Consortium has since discovered approximately 62,000 extra high-quality SNPs [15]. These SNPs have been used to develop a whole-genome cattle SNP genotyping microarray [16]. More recently, a novel higher-density whole-genome bovine SNP BeadChip, containing ~770,000 SNPs has being developed by Illumina [17].

With the availability of genome-wide dense marker maps and cost-effective genotyping methods, a novel genetic improvement method, called genomic selection, has been developed and is already revolutionising the cattle breeding industry. Genomic selection is a form of marker-assisted selection in which genetic markers covering the whole genome are used to estimate breeding values (genomic breeding values) [18]. However, since most of the SNPs present on the whole-genome cattle SNP genotyping microarrays commonly used, are not in genes and also because of the extent of linkage disequilibrium, SNPs associated with economically important traits, will most likely, not be involved directly in these traits. The identification of the causative genetic variants involved in the phenotypes of interest, remain a difficult task. It is therefore, crucial to develop strategies to pinpoint more rapidly causative genetic variants underlying phenotypes of interest.

The identification of these causative genetic variants, also known as quantitative trait nucleotides (QTNs) involves the mapping of quantitative trait loci (QTLs), the discovery of novel genetic markers in the QTL regions, the fine-mapping of QTLs and then the sequencing of candidate genes. This iterative process until recently was very time-consuming, but thanks to the availability of a large number of SNPs and to the relatively low-cost of whole-genome genotyping methodologies, the fine-mapping of QTL regions has now been expedited. In addition, the advent of novel sequencing technologies [19-23] offers now a new opportunity for the identification of QTNs, with the ability to partially or completely re-sequence mammalian genomes, in a relatively cost-effective manner, and to identify polymorphisms responsible for the traits of interest.

The genome of animals from many species has now been sequenced, including the genomes of several bulls [24-30]. For example, Eck *et al*. (2009) generated the first single cattle genome sequence by a next-generation sequencing method [24]. By sequencing the whole-genome sequence of one Fleckvieh bull, they discovered more than 2 million novel cattle SNPs. Even though sequencing costs plummeting, sequencing whole-genomes or large targeted regions is still too expensive for most laboratories.

A whole-transcriptome RNA sequencing (RNA-Seq) method has recently been developed to identify and quantify genes expressed in different tissues [31,32]. This method has also been used to identify polymorphisms in transcribed regions, in different species, including in cattle [33,34]. A transcriptomic-based sequencing approach offers a cheaper alternative to identify a large number of polymorphisms and possibly to discover QTNs.

In the present study, we performed a gene-based SNP discovery analysis in bovine *Longissimus thoraci*, using a whole-transcriptome sequencing approach. To our knowledge, this represents the first study done in bovine muscle. For this purpose, muscle samples from three different Limousin bulls were analysed. We have identified more than 34,000 putative SNPs, including more than 60% novel polymorphisms. To evaluate the accuracy of the SNPs detected, 48 putative SNPs were genotyped. One-hundred percent concordance was observed when a stringent SNP quality criterion was chosen. The RNA-Seq data and the collection of newly discovered coding SNPs improve the genomic resources available for cattle, especially for beef breeds. The large amount of variation present in genes expressed in Limousin *Longissimus thoracis*, especially the large number of non synonymous coding SNPs, may prove useful to study the mechanisms underlying the genetic variability of meat quality traits.

## Results and discussion

### RNA sequencing

To obtain a global view of the bovine *Longissimus thoracis* transcriptome at single-nucleotide resolution, poly(A)-enriched mRNA from three Limousin bull calves were retrotranscribed and subjected to high-throughput sequencing. The three RNA-Seq libraries were barcode-tagged and sequenced on one lane of an Illumina HiSeq2000 sequencer. Sequencing of cDNA libraries generated a total of 125,781,357 raw paired-end reads with a length of 100 bases, resulting in a total of 25 gigabases. The reads were de-multiplexed to assign reads to each sequenced sample according to its barcode index. Approximately 36 to 46 million paired-end reads were obtained for each library. Reads from each sample were then mapped back to the bovine reference transcriptome. We used the set of *Bos taurus* Ensembl transcripts v61 RefSeq genes as the reference transcriptome. This set contains transcripts for 22,915 known or novel genes but also pseudogenes. Based on mappings done using the Burrows—Wheeler Aligner (BWA) programme, 63% to 67% of the mapped reads were aligned properly paired (Table 1). Transcriptome contamination was negligible (0.19%-0.24%). A total of 19,752 transcripts (16,287 genes) were identified, with at least one paired-end read in all samples analysed. Similar RNA-Seq read mapping rate and the number of genes identified were obtained in other RNA-Seq bovine studies [33-38]. For example, Wickramasinghe *et al*. (2012) found that ~65% of the RNA-Seq reads they generated while sequencing the milk transcriptome mapped uniquely onto the bovine genome. They also found that ~17,000-19,000 genes were expressed in milk [35]. Baldwin and collaborators found, this time, by sequencing the rumen epithelium that ~71% of the reads mapped onto ~17,000 different genes [36].

**Table 1:** Summary of reads mapping to the bovine transcriptome

	**LIM1**	**LIM2**	**LIM3**	**Total**
Number of reads	43,176,380	36,125,981	46,478,996	125,781,357
Number of bases (in Gb)	8.72	7.30	9.39	25.41
Contamination	81,940	87,847	90,532	260,319
*E. coli*	275	351	290	916
PhiX	67,226	81,146	84,717	233,089
Yeast	14,439	6,360	5,525	26,324
%	0.19	0.24	0.19	0.21
Number of uniquely mapped paired-reads	27,122,319	24,132,331	29,640,240	80,894,890
%	62.82	66.80	63.77	64.31
Number of transcripts	18,356	18,417	18,493	19,752
Number of genes	15,189	15,242	15,303	16,287

Gene expression was normalised as paired-end reads mapped per million total uniquely mapped paired-end reads (FPKM). Amongst these transcripts, 14,298 (72%) were identified with more than 1 read per million in at least one library. Some transcripts were represented by many reads. Moreover, 50% of the reads mapped to only 77 transcript sequences and 90% mapped to 2,878 transcripts. The top twenty of these transcripts are shown in Table 2. Amongst these transcripts, several are associated with energy metabolism (cytochrome c oxidase subunit I, II and III, cytochrome b, ATP synthase subunit alpha, NADH dehydrogenase subunit I and NADH-ubiquinone oxidoreductase chain 3) or locomotion (alpha skeletal muscle actin, troponin T, myosin regulatory light chain 2, tropomyosin beta chain, myoglobin, myotilin, myosin 1 and myosin 7). These results were consistent with the physiological role of genes expected in the surveyed tissue.

**Table 2:** Top twenty transcripts with most assigned reads

**Gene ID^1^**	**Transcript ID**^**1**^	**Description**	**Chromosome**
ENSBTAG00000043561	ENSBTAT00000060569	cytochrome c oxidase subunit I	MT
ENSBTAG00000046332	ENSBTAT00000006534	actin, alpha skeletal muscle	28
ENSBTAG00000018369	ENSBTAT00000024444	myosin regulatory light chain 2, ventricular/cardiac muscle isoform	17
ENSBTAG00000005333	ENSBTAT00000007014	myoglobin	5
ENSBTAG00000018204	ENSBTAT00000009327	myosin-1	19
ENSBTAG00000043584	ENSBTAT00000060539	ATP synthase subunit a	MT
ENSBTAG00000012927	ENSBTAT00000017177	fructose-bisphosphate aldolase C-A	25
ENSBTAG00000021218	ENSBTAT00000028269	myosin regulatory light chain 2, skeletal muscle isoform	25
ENSBTAG00000043560	ENSBTAT00000060566	cytochrome c oxidase subunit 3	MT
ENSBTAG00000043556	ENSBTAT00000060549	cytochrome c oxidase subunit 2	MT
ENSBTAG00000013921	ENSBTAT00000018492	creatine kinase M-type	18
ENSBTAG00000010156	ENSBTAT00000013402	translationally-controlled tumor protein	12
ENSBTAG00000043550	ENSBTAT00000060567	cytochrome b	MT
ENSBTAG00000015214	ENSBTAT00000020243	carbonic anhydrase 3	14
ENSBTAG00000040053	ENSBTAT00000036426	myosin-7	10
ENSBTAG00000006419	ENSBTAT00000008420	troponin T, slow skeletal muscle	18
ENSBTAG00000011424	ENSBTAT00000015186	tropomyosin beta chain	8
ENSBTAG00000043568	ENSBTAT00000060547	NADH-ubiquinone oxidoreductase chain 3	MT
ENSBTAG00000007782	ENSBTAT00000010231	myotilin	7
ENSBTAG00000043558	ENSBTAT00000060571	NADH dehydrogenase subunit 1	MT

^1^ identifier from Ensembl.

MT, mitochondrial genome.

**Figure 1:**
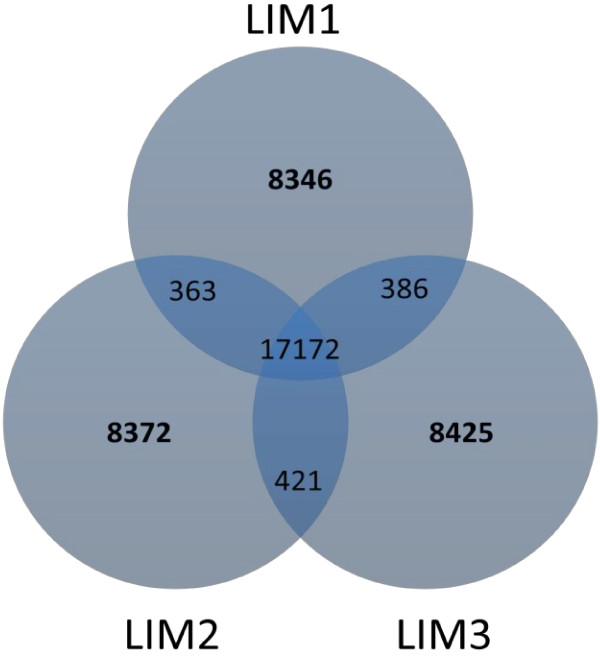
Unique and shared transcripts within the three muscle samples (Venn diagram).

To assess the consistency of gene expression profile measurements, the pairwise individual-to-individual Pearson correlation coefficient of the gene expression levels was calculated. The correlations were very high between individuals (r > 0.92) (Additional file 1: Table S1). The shared and unique presence of transcripts is shown in Figure 1. 17,172 (87%) of the transcripts were shared among the three samples. However, approximately 2% of the transcripts are only expressed in one sample.

### SNP discovery and annotation

For SNP calling, BWA was used to map the paired-reads from each sample to the bovine reference genome sequence. The SAM tools package was used for SNP discovery using stringent parameters (*e.g*. minimum coverage of 8 reads and mapping quality of 20). SAMtools can identify single base substitutions as well as small insertions and deletions; however, only SNPs were considered in the current analysis. In total 34,376 different SNP positions were detected with the RNA-Seq reads. Amongst these SNPs, 8,974 (26%) were homozygous in all three sequenced samples, corresponding presumably to differences between Limousin and the Hereford bovine whole-genome reference sequence [13]. A comparable number of SNPs were discovered by Canovas *et al*. (2010) using a similar total number of RNA-Seq reads (~118 millions reads). They identified ~100,000 SNPs located in genes expressed in milk samples from Holstein cows. However, only 33,045 SNPs (32%) were polymorphic within their seven Holstein cows [33].

In our study, we found that there were 30,998 bi-allelic SNPs mapping to coding regions, 38.6% of which were previously found and recorded in dbSNP. This high percentage of novel SNPs, even though there are currently more than 9 millions SNPs in the public SNP database dbSNP (version 133), suggests that a large fraction of the genetic variability present in Limousin cattle still remains to be discovered.

The proportion of transition substitutions were A/G, 36%, and C/T, 37%, compared to transversions A/C, 7%, G/T, 7%, A/T, 4% and C/G, 9%. This corresponds to a transition:transversion ratio of 2.65:1. The observed transition:transversion ratio is closed to the expected ratio (2:1) if all substitutions were equally likely.

Amongst these bi-allelic SNPs, 17,011 (55%) were found using Ensembl’s Variant Effect Predictor in a predicted coding region. 3,791 (22.23%) resulted in an amino acid change (nonsynonymous coding SNP; nscSNP) found in 2,438 different genes. The percentage of nonsynonymous changes in the coding region found in our study was lower compared to whole-genome [24-27] studies performed previously in cattle. For example, Kawahara-Miki *et al*. (2011) have reported up to 57.3% of nscSNPs in coding regions in the whole-genome of a single individual of the Japanese Kuchinoshima-Ushi native cattle breed [25]. They found 11,713 nscSNPs in 4,643 different genes. However, our results were similar to the rate found in another transcriptome-based study [34]. Huang and collaborators (2012) found 1,779 nscSNPs (in 1,369 genes) out of 6,941 coding SNPs (~25%) identified by sequencing the transcriptomes of leukocytes from three animals from three different breeds [34]. The broader gene coverage when sequencing DNA *versus* RNA might contribute to the discrepancy in the rate of nscSNPs found between whole-genome and transcriptome-based studies.

The deleterious effect of non-synonymous SNPs were analysed using the SIFT and PolyPhen algorithms. In order to use these programmes, sequences flanking the bovine nscSNPs were mapped onto the human genome and custom scripts were used to extract the human position orthologous to each bovine SNP position. We selected only bovines nscSNPs for which the two bases before and the two bases after the SNP exactly matched the human sequence. The human chromosomal position and the bovine alleles were combined to produce “pseudo human” variant positions and then used to query SIFT and PolyPhen. Using this conservative approach, we could retrieve the human “orthologous” position for 206 different bovine nscSNPs.

Using SIFT, we found that 90 different “pseudo human” coding variants were damaging. The three Limousin animals used were homozygous or heterozygous for 41 and 68 of these damaging SNPs, respectively. The difference between the number of SNPs found homozygous and heretozygous, reflects the fact that deleterious alleles are less likely to be homozygous. All three Limousin animals were homozygous for 17 damaging ncSNPs, including 13 SNPs with a genotype probability score above 20 (in all 3 samples) and 8 SNPs with a genotype probability score of 99 (in at least one sample).

Using PolyPhen-2, we found 69 different damaging “pseudo human” coding variants. 29 SNPs were homozygous and 52 SNPs heterozygous in at least one of the three Limousin samples. All Limousin animals were homozygous for 12 damaging nscSNPs, including 10 SNPs with a genotype probability score above 20 (in all 3 samples) and 6 SNPs with a genotype probability score of 99 (in at least one sample).

Fifty damaging nscSNPs were found by both SIFT and PolyPhen-2 algorithms, including 5 high-confidence nscSNPs for which all three Limousin animals are homozygous (Additional file 2: Table S2).

Gene Ontology analysis was performed with all genes containing nscSNPs. Out of the 2,438 genes, 1,092 (45%) were assigned to one or more GO annotations. In total 3,589, 2,892 and 8,172 GO terms were obtained for biological processes, cellular components and molecular functions, respectively. GO term analysis showed a significant enrichment of specific GO terms when comparing the annotations of SNP-containing genes against all unique transcripts from the bovine reference transcriptome. A summary of the classification of these genes into major biological process, cell component and molecular function categories is presented in Additional file 3: Table S3. Genes encoding proteins from the cytoskeleton and the extra-cellular matrix, or involved in cell cycle and cellular response are significantly over-represented. This finding might be explained by the high level of expression of these genes, that likely translates into greater sequence coverage and ultimately in a larger proportion of SNPs being identified in specific functional groups of genes. No significant enrichment in KEGG terms/pathways was found.

The positions of the 34,376 different SNPs predicted with the RNA-Seq reads were compared to the position on the UMD3.1 bovine genome assembly of know quantitative trait loci (QTLs) deposited in the public database AnimalQTLdb [39]. 32,631 SNPs were located in 3,855 different QTL regions (Additional file 4: Table S4). For example, 2,116 different SNPs are found in 16 QTL regions for meat tenderness score; whereas 14,560 SNPs are within 121 QTL regions for marbling score. QTLs were sorted into two groups (meat quality/muscle-related QTLs *versus* other QTLs) and the number of SNPs found in these two groups were counted. We then performed a Chi-squared test and found a significant difference (*P* = 0) in the number of SNPs between the two groups (Additional file 5: Table S5), suggesting an enrichment of SNPs in meat/muscle related QTLs. The high number of predicted SNPs located within known QTL regions, particularly in chromosomal regions harbouring QTLs for meat quality-related traits, indicates that the collection of SNPs found in the *Longissimus thoraci* transcriptome should allow the detection of candidate quantitative trait nucleotides responsible for the genetic variability of some of these traits.

### Selection of candidate SNPs and validation

To analyse the accuracy of RNA-Seq technology for SNP detection, a set of SNPs were selected for validation by genotyping. Non-synonymous SNPs are of particular interest because they are more likely to alter the structure and biological function of a protein, and therefore could be the causative mutations underlying important phenotypes. We therefore selected nscSNPs for validation. All suitable putative bi-allelic nscSNPs were evaluated with the Illumina ADT software. 2,452 nscSNPs (65%) with ADT score >0.6 passed the filtering step. In order to increase the probability of an *in silico* detected SNP being a truly polymorphic site, we selected nscSNPs already found in dbSNP. Finally, 48 putative nscSNPs detected in 38 genes were selected (Table 3).

**Table 3:** List of selected SNPs

**SNP**	**SNP ID^1^**	**SNP name**	**Ensembl transcript ID**	**Chromosome**	**Position**	**Reference allele**	**Alternative allele**
1	rs43270801	1_127257294	ENSBTAT00000044294	1	127257294	C	T
2	rs132988686	2_747896	ENSBTAT00000018496	2	747896	A	G
3	rs43299525	2_29938364	ENSBTAT00000038441	2	29938364	T	C
4	rs42982977	3_54421677	ENSBTAT00000055586	3	54421677	A	G
5	rs41255286	3_90246130	ENSBTAT00000015460	3	90246130	C	T
6	rs43360668	3_100666640	ENSBTAT00000003878	3	100666640	T	C
7	rs43414903	4_115404252	ENSBTAT00000028347	4	115404252	C	T
8	rs43447305	5_105538517	ENSBTAT00000009938	5	105538517	G	A
9	rs43484023	6_109946655	ENSBTAT00000060963	6	109946655	G	C
10	rs132780299	7_15769886	ENSBTAT00000013440	7	15769886	C	T
11	rs42722878	8_101639394	ENSBTAT00000001939	8	101639394	T	C
12	rs42722887	8_101642585	ENSBTAT00000001939	8	101642585	G	A
13	rs42722900	8_101645192	ENSBTAT00000001939	8	101645192	C	T
14	rs42722901	8_101645255	ENSBTAT00000001939	8	101645255	C	T
15	rs42306198	8_111749876	ENSBTAT00000008586	8	111749876	G	A
16	rs17870317	9_34687597	ENSBTAT00000038044	9	34687597	T	G
17	rs17870361	9_61258934	ENSBTAT00000015037	9	61258934	C	T
18	rs43626955	10_51842959	ENSBTAT00000007206	10	51842959	A	C
19	rs43626956	10_51843008	ENSBTAT00000007206	10	51843008	A	G
20	rs43626957	10_51843101	ENSBTAT00000007206	10	51843101	A	G
21	rs42284472	10_58147435	ENSBTAT00000008516	10	58147435	C	T
22	rs42748012	10_90111114	ENSBTAT00000016066	10	90111114	C	T
23	rs42738663	10_90126463	ENSBTAT00000016066	10	90126463	A	G
24	rs42311164	11_47748651	ENSBTAT00000005725	11	47748651	G	C
25	rs42613762	13_51391698	ENSBTAT00000025981	13	51391698	G	A
26	rs42555633	13_59146558	ENSBTAT00000002520	13	59146558	A	G
27	rs41255356	13_67838559	ENSBTAT00000018669	13	67838559	T	C
28	rs41712055	13_78093743	ENSBTAT00000026859	13	78093743	C	T
29	rs42929124	15_17647017	ENSBTAT00000004769	15	17647017	C	A
30	rs41774805	15_57309934	ENSBTAT00000006638	15	57309934	G	A
31	rs41720009	17_68389438	ENSBTAT00000053508	17	68389438	A	G
32	rs41905209	19_25255424	ENSBTAT00000061398	19	25255424	C	T
33	rs42803062	19_28474511	ENSBTAT00000044661	19	28474511	C	T
34	rs41930998	19_62070112	ENSBTAT00000009089	19	62070112	C	T
35	rs41969933	21_19283173	ENSBTAT00000014089	21	19283173	C	T
36	rs42013154	22_48725986	ENSBTAT00000019339	22	48725986	G	T
37	rs42016156	22_49203698	ENSBTAT00000045850	22	49203698	C	T
38	rs42015934	22_51561550	ENSBTAT00000007217	22	51561550	C	T
39	rs42451508	25_21535844	ENSBTAT00000008398	25	21535844	G	A
40	rs42174698	29_26367840	ENSBTAT00000002177	29	26367840	T	C
41	rs17871172	29_26368230	ENSBTAT00000002177	29	26368230	C	T
42	rs17871173	29_26368263	ENSBTAT00000002177	29	26368263	C	T
43	rs42188815	29_41795763	ENSBTAT00000012485	29	41795763	G	A
44	rs42188070	29_45033799	ENSBTAT00000023514	29	45033799	C	T
45	rs29024659	X_81605181	ENSBTAT00000003345	X	81605181	C	T
46	rs55617351	X_141005664	ENSBTAT00000029896	X	141005664	G	A
47	rs55617145	X_141005870	ENSBTAT00000029896	X	141005870	C	A
48	rs55617174	X_141005964	ENSBTAT00000029896	X	141005964	A	T

^1^ rs number from dbSNP.

The 48 selected SNPs were genotyped on the three original Limousin bull calves used for the RNA-Seq work, using llumina’s GoldenGate BeadXpress system. From the 48 SNPs that were genotyped, 11 SNP assays failed to work (23%), equivalent to a conversion rate of ~77%. We had 100% call rate for all remaining 37 SNPs with these three DNA samples (Table 4). A similarly low assay conversion rate was obtained in a recent SNP genotyping project using Illumina’s GoldenGate BeadXpress system and was due to failure in the synthesis of some of the oligonucleotides (unpublished data).

**Table 4:** Genotype comparison

			**RNASeq**			**RNASeq**			**BeadXPress**			
			**Genotypes**			**SNP quality score**			**Genotypes**			
**SNP**	**SNP ID**^**1**^	**SNP name**	**LIM1**	**LIM2**	**LIM3**	**LIM1**	**LIM2**	**LIM3**	**LIM1**	**LIM2**	**LIM3**	**Concordance (%)**
1	rs43270801	1_127257294	TT	TT	TT	35	35	38	--	--	--	
3	rs43299525	2_29938364	TT	TC	TC	4	4	3	TT	TC	TT	66.67
4	rs42982977	3_54421677	AG	GG	AG	46	13	14	--	--	--	
5	rs41255286	3_90246130	CC	CC	CT	99	99	99	CC	CC	CT	100.00
6	rs43360668	3_100666640	CC	CC	CC	14	9	6	CC	CC	CC	100.00
7	rs43414903	4_115404252	CC	CT	CC	71	99	92	CC	CT	CC	100.00
9	rs43484023	6_109946655	GG	GC	GG	5	5	5	GC	GG	CC	33.33
11	rs42722878	8_101639394	TC	CC	TT	3	3	4	TT	TC	TC	0.00
12	rs42722887	8_101642585	AA	GA	GA	14	26	3	AA	GA	GA	100.00
13	rs42722900	8_101645192	CC	CC	CT	8	4	55	CC	CC	CT	100.00
14	rs42722901	8_101645255	TT	CT	CT	6	15	4	TT	CT	CT	100.00
15	rs42306198	8_111749876	GG	GG	GA	79	82	99	GG	GG	GA	100.00
16	rs17870317	9_34687597	TG	TG	TG	3	3	3	TT	TT	TG	33.33
17	rs17870361	9_61258934	CT	CC	CT	54	20	21	CT	CC	CT	100.00
18	rs43626955	10_51842959	CC	CC	CC	99	99	99	CC	CC	CC	100.00
19	rs43626956	10_51843008	GG	GG	GG	99	99	99	GG	GG	GG	100.00
20	rs43626957	10_51843101	GG	GG	GG	79	76	76	GG	GG	GG	100.00
21	rs42284472	10_58147435	CC	CT	CC	14	11	8	--	--	--	
22	rs42748012	10_90111114	CT	CT	CC	19	59	65	CT	CT	CC	100.00
23	rs42738663	10_90126463	AG	AA	GG	72	16	23	AG	AG	GG	66.67
24	rs42311164	11_47748651	GC	CC	GC	29	24	77	GC	GG	GC	100.00
25	rs42613762	13_51391698	AA	AA	GG	4	8	6	AA	AA	GG	100.00
26	rs42555633	13_59146558	AG	AA	AG	3	4	49	AG	AG	AA	33.33
27	rs41255356	13_67838559	CC	TC	CC	4	6	4	TC	TT	CC	33.33
28	rs41712055	13_78093743	CT	TT	CC	30	21	24	TT	TT	--	
29	rs42929124	15_17647017	AA	AA	AA	23	20	23	--	--	--	
30	rs41774805	15_57309934	AA	GA	GA	8	47	68	AA	GA	GA	100.00
31	rs41720009	17_68389438	GG	GG	GG	31	43	34	AG	GG	GG	66.67
32	rs41905209	19_25255424	CT	CC	CC	22	10	54	CT	CT	CC	66.67
33	rs42803062	19_28474511	CC	CT	CT	52	33	62	CC	CT	CT	100.00
34	rs41930998	19_62070112	CC	CT	CT	14	6	4	--	--	--	
35	rs41969933	21_19283173	TT	CT	TT	5	5	5	TT	CT	TT	100.00
36	rs42013154	22_48725986	GT	GT	GG	99	99	99	GT	GT	GG	100.00
37	rs42016156	22_49203698	TT	TT	CC	48	24	27	TT	TT	CC	100.00
38	rs42015934	22_51561550	CC	CT	CC	29	6	35	CC	CT	CC	100.00
39	rs42451508	25_21535844	GA	GA	GA	39	31	70	GA	GA	GA	100.00
40	rs42174698	29_26367840	CC	CC	CC	52	40	70	CC	CC	CC	100.00
41	rs17871172	29_26368230	CC	CC	CT	56	47	30	CC	CC	CT	100.00
42	rs17871173	29_26368263	CT	TT	CT	99	37	99	--	--	--	
43	rs42188815	29_41795763	AA	AA	AA	99	99	99	--	--	--	
44	rs42188070	29_45033799	CC	CT	CC	26	26	14	CC	CT	CC	100.00
45	rs29024659	X_81605181	TT	TT	TT	26	26	29	TT	TT	TT	100.00
46	rs55617351	X_141005664	GA	GA	GA	3	3	3	GG	GG	GG	0.00
47	rs55617145	X_141005870	CA	CA	CA	3	3	3	CC	CC	CC	0.00
48	rs55617174	X_141005964	AT	AT	AT	3	3	3	TT	TT	TT	0.00

^1^ rs number from dbSNP.

A comparison between genotypes obtained by direct genotyping and predicted from the RNA-Seq data show 23 discrepancies (20%) (Table 4). A quick survey shows that discordant genotyping calls occur when genotypes have been predicted from the RNA-Seq data with a low probability (score below 20). Only two discrepancies (1.8%) remained when RNA-Seq-based genotypes having at least a probability score of 20 were selected, and no discrepancies were observed when using the highest probability threshold (score of 99). It is important to point out that the RNA-Seq-based genotypes were derived from cDNA sequences whereas the genotypes produced by genotyping were obtained from DNA samples. The two discrepancies seen after filtering with a probability score above 20 (SNP26 AG *versus* AA and SNP31 GG *versus* AG; RNA-Seq-based genotype *versus* BeadXPress-based genotype) could therefore possibly be true differences between RNA and corresponding DNA samples, due to A-to-I (G) RNA editing (*e.g*. [40] and allele-specific expression [41], respectively.

The SNP discovery analysis was performed initially without filtering the individual genotypes derived from the RNA-Seq data. Following on our validation study, we further filtered the identified SNPs, using this time the highest genotype probability score. We selected SNPs for which at least one individual had a heterozygous or the alternative homozygous genotype, with a probability score equal to 99. We detected 8,407 different high-confidence SNPs among 3,867 transcripts. Amongst these SNPs, 1,966 (23%) were homozygous in all three sequenced samples; 8,199 (97%) were bi-allelic SNPs; 3,123 (37%) were previously found in dbSNP; 6,158 (73%) were found in coding regions and 1,242 (18%) resulted in an amino acid change (in 948 different genes). A list of the high-confidence SNPs is available, as an additional file to this manuscript (Additional file 6: Table S6).

### Population genetics screens

To test the usefulness of the identified SNPs, the 48 selected nscSNPs were assessed by genotyping a total of 90 bovine samples (including the three Limousin samples used for the RNA-Seq work) representing the 9 major breeds used in France, an African taurine breed (Watusi), and two other *Bovinae* species (European bison and Greater Koudou).

As reported above, 8 SNP assays failed to work in all samples. SNP call rate ranged from 55% (rs42555633) to 100%, whereas the call rate for bovine DNA samples ranged from 93% to 98%.

The majority (95%) of the selected SNPs with working assays, generated data with the European bison and the Greater Koudou samples (35/37 and 27/37 SNPs, respectively) (Table 5). This could be expected since the markers were developed from (conserved) intra-genic regions. Only 3 SNPs exhibited polymorphisms in these two outcross species (2 SNPs in European bison and 2 SNPs in Greater Koudou). However, due to the small sample size (n = 1), this number is likely to be downwardly biased and a higher proportion of SNPs may in fact be polymorphic and therefore prove useful in these species. As expected from the phylogenetics of these species, the proportions of working SNPs were lower in the Greater Koudou than in the European bison.

**Table 5:** Details and allele frequencies of SNPs in the nine French cattle breeds, and genotypes in the three other samples

**SNP**	**SNP ID^1^**	**Chromosome**	**Position^2^**	**Gene**	**Alleles**				**Frequency (allele 1)**						**Genotype**			
					**1/2**		**AUB**	**BLA**	**CHA**	**HOL**	**LIM**	**MAN**	**MON**	**NOR**	**SAL**	**WAT**	**BIS**	**KOU**
1	rs43299525	2	29,938,364	ENSBTAT00000038441	T/C		0.18	0	0.23	0.28	0.23	0.17	0.45	0.25	0.41	T/T	T/T	T/T
2	rs41255286	3	90,246,130	ENSBTAT00000015460	C/T		0.23	0.14	0.18	0.36	0.27	0.42	0.45	0.67	0.14	G/G	G/G	G/G
3	rs43360668	3	100,666,640	ENSBTAT00000003878	T/C		0.09	1	1	0.04	0.14	0.17	0.18	0.25	0.04	G/G	G/G	G/G
4	rs43414903	4	115,404,252	ENSBTAT00000028347	C/T		0.18	0.09	0.27	0.09	0.14	0.08	0	0.25	0.09	C/C	C/C	C/C
5	rs43484023	6	109,946,655	ENSBTAT00000060963	G/C		0.18	0.32	0.36	0.14	0.45	0.25	0.68	0.17	0.09	G/C	C/C	C/C
6	rs42722878	8	101,639,394	ENSBTAG00000020243	T/C		0.18	0.04	0.36	0.04	0.59	0.25	0.41	0.17	0.04	T/C	C/C	C/C
7	rs42722887	8	101,642,585	ENSBTAG00000020244	G/A		0.27	0.09	0.36	0.04	0.59	0.25	0.41	0.17	0.04	G/A	G/G	G/G
8	rs42722900	8	101,645,192	ENSBTAG00000020245	C/T		0.04	0.04	0.14	0	0.41	0.25	0.27	0.08	0.04	C/C	C/C	C/C
9	rs42722901	8	101,645,255	ENSBTAG00000020246	C/T		0.24	0.09	0.35	0.04	0.59	0.25	0.41	0.17	0.04	C/T	C/C	C/C
10	rs42306198	8	111,749,876	ENSBTAT00000008586	G/A		0	0.04	0.09	0.04	0.18	0	0	0.08	0.04	G/G	G/G	
11	rs17870317	9	34,687,597	ENSBTAT00000038044	T/G		0.33	0.32	0.45	0.45	0.41	0.50	0.32	0.67	0.32	T/T	T/T	T/T
12	rs17870361	9	61,258,934	ENSBTAT00000015037	C/T		0.24	0.14	0.04	0.73	0.14	0.17	0.32	0	0.04	C/C	C/C	
13	rs43626955	10	51,842,959	ENSBTAT00000007206	A/C		0.36	0.41	0.23	0.14	0.82	0.92	0.54	0.17	0.68	A/C	C/C	C/C
14	rs43626956	10	51,843,008	ENSBTAT00000007207	A/G		0.36	0.41	0.23	0.14	0.82	0.92	0.54	0.25	0.68	A/G	G/G	A/G
15	rs43626957	10	51,843,101	ENSBTAT00000007208	A/G		0.59	0.50	0.32	0.27	0.95	1	0.54	0.25	0.77	A/G	G/G	
16	rs42748012	10	90,111,114	ENSBTAT00000016066	C/T		0.64	0.50	0.68	0.77	0.50	0.33	0.86	0.33	0.68	T/T	C/C	C/C
17	rs42738663	10	90,126,463	ENSBTAT00000016067	A/G		0.36	0.50	0.32	0.23	0.50	0.67	0.14	0.67	0.32	A/A	G/G	G/G
18	rs42311164	11	47,748,651	ENSBTAT00000005725	G/C		0.27	0.36	0.23	0.14	0.32	0.67	0.27	0.42	0.50	G/G	C/C	C/C
19	rs42613762	13	51,391,698	ENSBTAT00000025981	G/A		0.73	0.95	0.70	0.23	0.68	0.92	0.54	0.58	0.86		G/A	G/A
20	rs41255356	13	67,838,559	ENSBTAT00000018669	T/C		0.36	0.32	0.73	0.23	0.54	0.08	0.27	0.83	0	T/T	T/C	
21	rs41774805	15	57,309,934	ENSBTAT00000006638	G/A		0.27	0.45	0.27	0.27	0.64	0.33	0.36	0.50	0.50	G/G	G/G	G/G
22	rs41720009	17	68,389,438	ENSBTAT00000053508	A/G		0.41	0.41	0.54	0.27	0.23	0.08	0.23	0.25	0.23	G/G	A/A	
23	rs41905209	19	25,255,424	ENSBTAT00000061398	C/T		0.14	0	0.14	0.59	0.09	0.17	0	0.08	0	C/C	C/C	C/C
24	rs42803062	19	28,474,511	ENSBTAT00000044661	C/T		0.36	0.68	0.59	0.23	0.59	0.08	0.73	0.58	0.54	C/C		
25	rs41969933	21	19,283,173	ENSBTAT00000014089	C/T		0.77	0.68	0.86	0.36	0.77	0.67	0.82	0.58	0.86	C/C	C/C	T/T
26	rs42013154	22	48,725,986	ENSBTAT00000019339	G/T		0.27	0.04	0.14	0.09	0.36	0.25	0.23	0	0.14	G/G	G/G	
27	rs42016156	22	49,203,698	ENSBTAT00000045850	C/T		0.56	0.50	0.65	0.32	0.82	1	0.86	0.58	0.68	C/C	C/C	
28	rs42015934	22	51,561,550	ENSBTAT00000007217	C/T		0.23	0.04	0.14	0.04	0.04	0.17	0.18	0.33	0.09	C/C	C/C	C/C
29	rs42451508	25	21,535,844	ENSBTAT00000008398	G/A		0.14	0.09	0.27	0.04	0.31	0.83	0.41	0.33	0.27	G/G	G/G	G/G
30	rs42174698	29	26,367,840	ENSBTAG00000001660	T/C		0.36	0.50	0.91	0.54	0	0.50	0.04	0	0.41		C/C	C/C
31	rs17871172	29	26,368,230	ENSBTAG00000001661	C/T		0	0.04	0	0	0.04	0	0	0.08	0	C/T	C/C	C/C
32	rs42188070	29	45,033,799	ENSBTAT00000023514	C/T		0.14	0.09	0.54	0.14	0.18	0.25	0.18	0.25	0.27	C/C	C/C	C/C
33	rs29024659	X	81,605,181	ENSBTAG00000002585	C/T												C/C	C/C
34	rs55617351	X	141,005,664	ENSBTAT00000029896	G/A											G/G	A/A	A/A
35	rs55617145	X	141,005,870	ENSBTAT00000029897	C/A											C/C	A/A	C/C
36	rs55617174	X	141,005,964	ENSBTAT00000029898	A/T											T/T	T/T	
Mean MAF (autosomes)							0.25	0.22	0.25	0.19	0.27	0.20	0.26	0.24	0.20			

^1^ rs number from dbSNP.

^2^ Position on the UMD3.1 cattle genome assembly.

*AUB*, Aubrac, *BLA*, Blonde d’Aquitaine, *CHA*, Charolais, *HOL*, Holstein, *LIM*, Limousin, *MAN*, Maine Anjou, *MON*, Montbéliarde, *NOR*, Normande, *SAL*, Salers, *WAT*, Watusi, *BIS*, European bison, *KOU*, Greater Koudou.

The observed allele frequencies for the all autosomal SNPs with a SNP call rate above 92% are shown in Table 5, for each cattle population. All autosomal SNPs had a minor allele frequency (MAF) >= 0.04 in all populations, with the exception of 13 SNPs which had a fixed allele in at least one population. The highest SNP MAF observed was 0.50. The mean MAF for all autosomal markers ranged from 0.19 (HOL) to 0.27 (LIM).

The observed heterozygosities, expected heterozygosities under HWE for the observed population allele frequencies, and significance level for the test for departures from HWE for each autosomal SNP, are shown in Additional file 7: Table S7. All these markers were in agreement with HWE (*P* = 0.001). The mean observed heterozygosity estimated for all autosomal markers, for each population ranged from 0.259 (+/− 0.176) to 0.386 (+/− 0.230). The mean observed heterozygosities in our populations were similar to values estimated in previous studies, including a study that used a whole-genome SNP panel to characterise the genetic diversity of several French cattle breeds [42,43].

The overall genetic differentiation among breeds was moderate (*F*_ST_ = 10.9% and *G*_ST_ = 9.86%) but highly significant from zero (unpublished data). This genetic differentiation among breeds implies that approximately 90% of the total genetic variation was explained by individual variability. A similar genetic differentiation was previously reported in a study carried out on French breeds, using microsatellite markers [44].

The exact test for population differentiation based on allele frequency variations shows that all breeds tested were significantly different from each other (*P* < 0.0001, unpublished data). Genetic distances between breeds were measured by pair-wise *F*_ST_ as shown in Additional file 8: Table S8. The HOL breed was the most differentiated one. The largest similarity was detected between BLA and SAL animals (*F*_ST_ = 0.0011). These results were in agreement with a previous study that analysed the genetic relationships between BLA, HOL, LIM and SAL populations [44]. Gautier and collaborators found in their study that HOL is also the most differentiated breed; however they found that AUB and LIM animals shared the smallest *F*_ST_ (*F*_ST_ = 0.0353) [42]. This discrepancy with our findings might mostly be due to the LIM population they surveyed. Since their study included US LIM animals, it is possible that these LIM animals were not pure-bred animals, unlike the LIM animals we used.

The degree of genetic differentiation among the breeds studied and the high levels of significance for the between-population *F*_ST_ estimates indicate a relatively low gene flow between these breeds.

**Figure 2:**
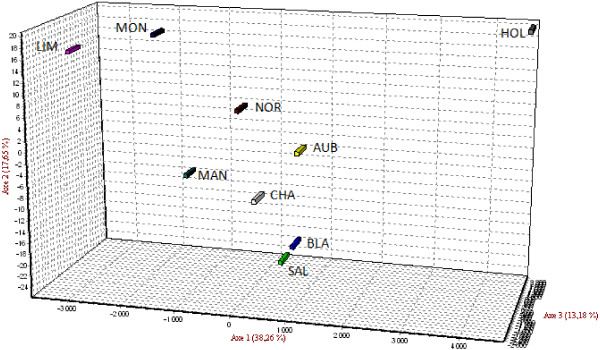
**Principal component analysis.** Per cent value in each axis indicates contribution to the total genetic variation.

Principal component analysis was performed including all animals and all autosomal loci using allele frequencies to summarise breed relationships. The analysis indicates a clear separation between the nine populations (Figure 2), but also some variability within each breed (Additional file 9: Figure S1). A total of approximately 69% of the variance accounted for the first three dimensions of the PCA.

### Functional candidate mutations

The discovered coding SNPs, especially the 8,407 high-confidence SNPs may have a direct functional effet and some of them may be involved in the genetic variability of meat quality traits.

Among the high-confidence non synonymous coding SNPs, we have identified a single polymorphism resulting in a premature stop codon. SNP rs135279925 (ENSBTAT00000007104:c.1093C>T) is located within the 10^th^ and last exon of *CD46*, a membrane cofactor protein. This variant leads to a three amino acid shortened protein. None of the sampled animals were homozygous for this mutation. The corresponding bovine gene (ENSBTAG00000005397) has three known different transcripts encoding 343, 361 and 367 amino acid long proteins. The nscSNP modifies the longest bovine protein version; however, as the last three amino acids are not conserved within the bovine proteins or between species, the polymorphism is unlikely to have a functional impact.

We also found among the high-confidence nscSNPs, the previously reported F94L mutation (rs110065568: BTA2 g.6213980C>A) in the *growth differentiation factor 8* (*GDF8*). *GDF8* is a known muscle growth factor inhibitor commonly known as *myostatin* (*MSTN*). This gene has been identified as the gene responsible for the double-muscling phenotype in cattle [45-47]. Numerous mutations in *MSTN* have been described in many breeds that cause muscle hypertrophy [45-51], including a non synonymous amino acid substitution (F94L) in a region of the protein known to be the inhibitory domain of the MSTN propeptide [52]. Limousin cattle are not considered a double-muscled breed, however genotyping of the SNP rs110065568 has shown that the A allele is present at high frequency [48-50,53]. Interestingly, the three sampled animals were homozygous for this mutation. Several studies have shown that the F94L mutation is associated with increased muscle mass, carcass yield, meat tenderness and with a reduction of collagen content in Limousin and Limousin-cross cattle [54-56]. The high frequency of the mutant allele in Limousin most likely reflects the effects of selection for increased muscle mass.

We found among the high-confidence polymorphisms a nscSNP in another bovine gene known to be involved in meat quality traits: the mutation A127S (rs109995479: BTA2 g.107515456C>A) in the *protein kinase adenosine monophosphate-activated α3-subunit* (*PRKAG3*). Studies have shown that mutations in the porcine *PRKAG3* affect the glycogen content in muscle, and consequently, ultimate pH, meat colour, water-holding capacity, drip loss, tenderness and cooking loss [57,58]. Because of the association of this gene with meat quality traits, polymorphism screens in the bovine *PRKAG3* have also been performed and several non synonymous SNPs have been identified, including SNP rs109995479 [59-61]. Associations between another polymorphism within *PRKAG3* and meat colour traits and cooking loss have been found in cattle [62]. It will be therefore interesting to test the effects of SNP rs109995479. This nscSNP is located within a region of the gene highly conserved in mammals; however, it is not located within any of the cystathione βsynthetase domains, where the two mutations with the highest phenotypic effects (I199V and R200Q) have been found, in pig.

In addition, we identified several polymorphisms in new candidate genes for several meat quality-related traits. For example, we found a high-confidence non synonymous coding SNP (rs109813896: BTA1 g.134130474G>C) in the gene encoding the *mitochondrial propionyl-coA carbolylase beta subunit* (*PCCB*), which is involved in the catabolism of propanoate, an important intermediate in the metabolism of several amino acids. Yang and collaborators [63] have shown that a polymorphism in *PCCB* is associated with fat weight, in pig. Interestingly, the bovine *PCCB* gene lies within a QTL region for fat thickness at the 12^th^ rib [64]. *PCCB* could therefore be a good candidate gene for this trait.

We also found seven high-confidence nscSNPs (including previously discovered SNPs: rs136458240, rs211315064 and rs209586352) in the gene encoding the *heparin sulfate proteoglycan 2* (*HSPG2*, ENSBTAG00000017122). This gene encodes a large proteoglycan that is a component of the extracellular matrix. Choi and collaborators [65] found an association between a polymorphism within this gene and marbling score, in pig. The bovine *HSPG2* gene is located within a marbling score QTL [66] and could therefore be a good candidate for this phenotype.

## Conclusions

Our results represent the first study of gene-based SNPs discovered using RNA-seq in bovine muscle. Our results show that RNA-Seq is a fast and efficient method to identify SNPs in coding regions and we identified more than 34,000 putative SNPs (including more than 8,000 high-confidence SNPs). More than 60% of these SNPs are completely novel. The high percentage of validation confirms the utility of the SNP-mining process and the stringent quality criteria for distinguishing sequence variations from sequencing errors or artifacts introduced during the preparation of the cDNA libraries. The RNA-Seq data and the collection of newly discovered coding SNPs improve the genomic resources available for cattle, especially for beef breeds. The large amount of variation present in genes expressed in Limousin *Longissimus thoracis*, especially the large number of non synonymous coding SNPs, may prove useful to study the mechanisms underlying the genetic variability of meat quality traits. The coding SNPs could also be used to study allele-specific gene expression.

Our approach could be further improved in order to reduce the cost of SNP discovery and validation. Higher multiplexing of cDNA libraries prior to sequencing, would reduce sequencing cost while still allowing SNP discovery and genotype assignment. With continued improvements in next-generation DNA sequencing technologies, throughput will increase while sequencing costs are expected to decrease. When relevant tissue samples are available, it will soon be reasonable to directly perform association studies using a genotyping RNA-Seq-based approach.

## Methods

### Animal ethics

All animal experimentation complied with the French Veterinary Authorities’ rules. No ethics approval was required by a specific committee, as the selected animals were not animals bred for experimental reasons.

#### Animals and tissue samples

The study was conducted with three Limousin bull calves from a large study on the genetic determinism of beef and meat quality traits [67]. The three bull calves were not closely related to one another (for at least 4 generations) were fattened in a single feedlot and fed *ad libidum* with wet corn silage. They were humanely slaughtered in an accredited commercial slaughterhouse when they reached 16 months. *Longissimus thoracis* (LT) muscle samples were dissected immediately after death and tissue samples were snap frozen in liquid nitrogen and stored at −80°C until analysis.

### RNA isolation and sequencing

After transfer to ice-cold RNeasy RLT lysis buffer (Qiagen, Courtaboeuf, France), LT tissue samples were homogenised using a Precellys tissue homogeniser (Bertin Technologie, Montigny-le-Bretonneux, France). Total RNA was isolated using RNeasy Midi columns (Qiagen) and then treated with RNAse-free DNase I (Qiagen) for 15 min at room temperature according to the manufacturer’s protocols. The concentration of total RNA was measured with a Nanodrop ND-100 instrument (Thermo Scientific, Ilkirch, France) and the quality was assessed with an RNA 6000 Nano Labchip kit using an Agilent 2100 Bioanalyzer (Agilent Technologies, Massy, France). All three samples had an RNA Integrity Number (RIN) value greater than eight.

The mRNA-Seq libraries were prepared using the TruSeq RNA Sample Preparation Kit (Illumina, San Diego, CA) according to the manufacturer’s instructions. Briefly, Poly-A containing mRNA molecules were purified from 4 μg total RNA of each sample using oligo(dT) magnetic beads and fragmented into 150–400 bp pieces using divalent cations at 94°C for 8 min. The cleaved mRNA fragments were converted to double-stranded cDNA using SuperScript II reverse transcriptase (Life Technologies, Saint Aubin, France) and primed by random primers. The resulting cDNA was purified using Agencourt AMPure® XP beads (Beckman Coulter, Villepinte, France). Then, cDNA was subjected to end-repair and phosphorylation and subsequent purification was performed using Agencourt AMPure® XP beads (Beckman Coulter). These repaired cDNA fragments were 3′-adenylated producing cDNA fragments with a single ‘A’ base overhung at their 3′-ends for subsequent adapter-ligation. Illumina adapters containing indexing tags were ligated to the ends of these 3′-adenylated cDNA fragments followed by two purification steps using Agencourt AMPure® XP beads (Beckman Coulter). Ten rounds of PCR amplification were performed to enrich the adapter-modified cDNA library using primers complementary to the ends of the adapters. The PCR products were purified using Agencourt AMPure® XP beads (Beckman Coulter) and size-selected (200 ± 25 bp) on a 2% agarose Invitrogen E-Gel (Thermo Scientific). Libraries were then checked on an Agilent Technologies 2100 Bioanalyzer using the Agilent High Sensitivity DNA Kit and quantified by quantitative PCR with the QPCR NGS Library Quantification kit (Agilent Technologies). After quantification, tagged cDNA libraries were pooled in equal ratios and a final qPCR check was performed post-pooling. The pooled libraries were used for 2×100 bp paired-end sequencing on one lane of the Illumina HiSeq2000 with a TruSeq SBS v3-HS Kit (Illumina). After sequencing, the samples were demultiplexed and the indexed adapter sequences were trimmed using the CASAVA v1.8.2 software (Illumina).

#### Mapping reads to reference transcriptome and gene expression counts

The *Bos taurus* reference transcriptome was downloaded from Ensembl (version 63, Bos_taurus.Btau_4.0.63.cdna.all.fa). To align the reads back to the assembled reference transcriptome the BWA programme (version 0.5.9-r16) was used [68]. Reads were mapped for each sample separately to the assembled transcriptome. The BWA default values were used for mapping. Properly paired reads with a mapping quality of at least 30 (−q = 30) were extracted from the resulting BAM file using SAMtools [69] for further analyses. Properly paired is defined as both left and right reads mapped in opposite directions on the same transcript at a distance compatible with the expected mean size of the fragments (<500-bp). Custom scripts were developed to identify paired-reads mapping to single locations and with the expected distance. Read pairs mapping to separate chromosomes were discarded for the present study. Transcriptome contamination was assessed by mapping with BWA reads on a sequence library, containing *E. coli*, phiX and yeast genome sequences. The number of paired-reads uniquely aligning to transcribed regions of each transcript was calculated for all genes in the annotated transcriptome. The transcript paired-read count was calculated as the number of unique paired-reads that aligned within the exons of each transcript, based on the coordinates of mapped reads. The expression level of each gene was calculated in FPKM (fragments per kilobase per million sequenced reads) using a custom script based on Tapnel *et al.* (2010) [70].

### Polymorphism identification

BWA was also used to map reads onto the bovine genome reference sequence (version UMD3.1, [71]. Only reliable properly paired BWA mapped reads were considered for Single Nucleotide Polymorphism (SNP) calling. Indels were not considered because alternative splicing impedes reliable indel discovery. SNPs were called using the SAMtools software package. Genotype likelihoods were computed using the SAMtools utilities and variable positions in the aligned reads compared to the reference were called with the BCFtools utilities [72]. SNPs were called only for positions with a minimal mapping quality (−Q) of 30, a minimum coverage (−d) of 4 and a maximum read depth (−D) of 10,000,000.

### Functional annotation of detected SNPs

The functional effect of the newly discovered SNPs on known transcripts were analysed using Ensembl’s Variant Effect Predictor v2.5, following local installation [73].

The deleterious effect of non-synonymous SNPs were analysed using the SIFT (Sorting Intolerant From Tolerant; http://sift.bii.a-star.edu.sg; [74] and PolyPhen-2 (Polymorphism Phenotyping 2; http://genetics.bwh.harvard.edu/pph2/; [75] programmes. In order to use these two programmes, sequences flanking the bovine nscSNPs were mapped onto the human genome (version GRCh37/hg19) using MegaBLAST [76] and custom scripts were used to extract the human position orthologous to each bovine SNP position. The human chromosomal position and the bovine alleles were then used to query SIFT and PolyPhen. Default settings were used for both programmes. We refered to damaging SNPs, SNPs that were identified as damaging and not tolerated, using PolyPhen-2 and SIFT, respectively.

In order to evaluate whether SNP-containing genes were significantly enriched for specific gene ontology (GO) terms and KEGG pathways compared to all annotated bovine genes, gene enrichment analyses were conducted using the FATIGO tool of the online software suite Babelomics (http://babelomics.bioinfo.cipf.es; [77]. Genes were assigned their Ensembl identities as input for Babelomics. Only one copy of each gene was used. Default parameter settings were used for the analysis. Statistical assessment of annotation differences between the two sets of sequences (SNP-containing genes *versus* all the other bovine genes) was carried out for each FATIGO analysis, using the Fisher Exact Test with correction for multiple testing.

#### Selection of candidate SNPs for genotyping assay

After SNP detection, *in silico* evaluation of candidate SNPs was carried out to select a panel of candidate SNPs for validation. SNP selection was based on the results from the Illumina Assay Design Tool. The SNP score from the Illumina Assay Design Tool (referred to as the Assay Design Score/ADS) utilises factors including template GC content, melting temperature, sequence uniqueness, and self-complementarity to filter the candidate SNPs prior to further inspection. The Assay Design Score (assigned between 0 and 1) is indicative of the ability to design suitable oligos within the 60 bp up/down-stream flanking regions, and the expected success of the assay when genotyped with the Illumina GoldenGate chemistry. Following the Illumina guidelines, all SNPs with a score below 0.4 should be discarded; SNPs with a score above 0.4 accepted, with SNPs scoring above 0.6 being used preferentially. SNP flanking sequences were retrieved and only SNP sequences with unambiguous 121 bases (60 bases up/down-stream of each SNP position) were submitted to Illumina to assess the design quality. SNPs with ADS showing a quality score above of 0.6 were retained for analysis.

### SNP validation by high-throughput genotyping

Ninety bovine DNA samples were genotyped for each selected SNP using Illumina’s GoldenGate assay. These samples include 11 Aubrac (AUB), 11 Blonde d’Aquitaine (BLA), 11 Charolais (CHA), 11 Holstein (HOL), 11 Limousin (LIM), 11 Montbéliard (MON), 11 Salers (SAL), 6 Maine-Anjou (MAN), 6 Normande (NOR) and 1 Watusi (WAT) animals. These animals were not closely related to one another (for at least 4 generations) according to genealogical records from the French Centre de Traitement de l’Information Génétique (INRA, Jouy-en-Josas, France). To assess the utility of developed markers in related species, two *Bovinae* species; the European bison (BIS, *Bison bonasus*) and a more distantly related species; the Greater Koudou (KOU, *Tragelaphus strepsiceros)* were also genotyped. Blood samples were collected at the Parc du Rénou Zoo (Le Vigen, France). Genomic DNA was extracted from whole-blood or semen samples using the Qiasymphony SP robotic system and DNA Midi kit (Qiagen). Quality of DNA was checked using a Nanodrop ND-100 spectrophotometer (Thermo Scientific) and quantity was estimated with Quant-iT Picogreen dsDNA kit (Life Technologies) on an ABI 7900HT (Life Technologies). All DNA samples were standardised to 50 ng/μL. All animal manipulations were done according to good animal practice as defined by the French Veterinary Authorities.

High-throughput genotyping reactions were performed using Illumina’s GoldenGate BeadXpress system, according to the manufacturer’s protocol. Oligonucleotides were designed, synthesised, and assembled into a custom oligo pooled assay (OPA) by Illumina. Automatic allele calling for each SNP was accomplished with the GenomeStudio software (Illumina). All genotypes were manually checked and re-scored if any errors in calling homozygous or heterozygous clusters were evident. Genotype calls were exported in spreadsheets from the GenomeStudio data analysis software for further analysis.

### Population genetics analyses

Genetic diversity parameters within each population were calculated using the GENETIX 4.05.2 software package [78]. Tests for deviation from Hardy–Weinberg equilibrium were performed by the GENEPOP 3.4 software [79], using the exact test of Guo and Thompson (1992) [80]. Genetic differentiation among and within the populations was estimated based on *F*-statistics (*F*_ST_) according to Weir and Cockerham (1984) [81]) using the GENEPOP and GENETIX software packages. Test for population differentiation was performed as implemented in GENEPOP. The Reynolds genetic distance (*D*_R_) was calculated for each pair of populations based on allele frequencies [82] using the GENETIX software. Principal component analysis (PCA) was performed using the GENETIX programme from allele doses for each individual.

### Data availability

The sequencing data have been submitted to the European Nucleotide Archive (accession number ERP002220).

## Competing interests

The authors declare that they have no competing interests.

## Authors’ contributions

AD carried out the bioinformatic analysis, under the supervison of CK. DE performed the RNA-Seq experiment. BW and MB contributed to the data analyses. DE and FM performed the SNP genotyping. CM prepared the RNA samples. DR conceived the study, analysed the data and drafted the manuscript. All authors read and approved the final manuscript.

## Supplementary Material

Additional file 1: Table S1 Pearson correlation coefficient between individuals. Click here for file

Additional file 2: Table S2 List of damaging SNPs predicted by SIFT and PolypPhen-2. Click here for file

Additional file 3: Table S3 Enrichment of SNP-containing contigs in GO terms. Click here for file

Additional file 4: Table S4 List of putative SNPs located within known QTL regions. Click here for file

Additional file 5: Table S5 Chi-squared test details. Click here for file

Additional file 6: Table S6 List of the high-confidence SNPs and annotation. Click here for file

Additional file 7: Table S7 Details on the observed and expected heterozygosities. Click here for file

Additional file 8: Table S8 Genetic differentiation (*F*_ST_) between pairs of cattle populations (above the diagonal) and Reynold’s genetic distance (*D*_R_) between pairs of cattle populations (below diagonal) as observed in this study. Click here for file

Additional file 9: Figure S1 Principal Component Analysis. Per cent value in each axis indicates contribution to the total genetic variation. Click here for file
