# Supplementary material for: Gene-based single nucleotide polymorphism discovery in bovine muscle using next-generation transcriptomic sequencing
Source: BMC Genomics. 2013 May 7;14:307. doi: 10.1186/1471-2164-14-307 (PMC3751807; doi:10.1186/1471-2164-14-307)
Supplement: Additional file 1: Table S1 — Pearson correlation coefficient between individuals. [file 1471-2164-14-307-S1.docx]

**Table S1**

LIM2 LIM3

LIM1 0.9232 0.9772

LIM2 0.9358
