# Supplementary material for: Gene-based single nucleotide polymorphism discovery in bovine muscle using next-generation transcriptomic sequencing
Source: BMC Genomics. 2013 May 7;14:307. doi: 10.1186/1471-2164-14-307 (PMC3751807; doi:10.1186/1471-2164-14-307)
Supplement: Additional file 7: Table S7 — Details on the observed and expected heterozygosities. [file 1471-2164-14-307-S7.docx]

**Table S7**

**AUB BLA CHA HOL LIM MAN MON NOR SAL**

**SNP SNP ID *H*_o_ *H*_e_ HWE *H*_o_ *H*_e_ HWE *H*_o_ *H*_e_ HWE *H*_o_ *H*_e_ HWE *H*_o_ *H*_e_ HWE *H*_o_ *H*_e_ HWE *H*_o_ *H*_e_ HWE *H*_o_ *H*_e_ HWE *H*_o_ *H*_e_ HWE**

1 rs43299525 0.182 0.298 0.556 0 0 1 0.455 0.351 1 0.545 0.397 0.793 0.455 0.351 1 0.333 0.278 1 0.727 0.496 0.427 0.500 0.375 1 0.636 0.483 0.793

2 rs41255286 0.455 0.351 1 0.273 0.236 1 0.364 0.298 1 0.364 0.463 0.811 0.182 0.397 0.217 0.500 0.486 1 0.545 0.496 1 0.333 0.444 1 0.273 0.236 1

3 rs43360668 0.182 0.165 1 0 0 1 0 0 1 0.091 0.087 1 0.273 0.236 1 0.333 0.278 1 0.364 0.298 1 0.500 0.375 1 0.091 0.087 1

4 rs43414903 0.364 0.298 1 0.182 0.165 1 0.545 0.397 0.793 0.182 0.165 1 0.273 0.236 1 0.167 0.153 1 0 0 1 0.500 0.375 1 0.182 0.165 1

5 rs43484023 0.182 0.298 0.556 0.455 0.434 1 0.364 0.463 0.811 0.273 0.236 1 0.545 0.496 1 0.167 0.375 0.545 0.273 0.434 0.464 0.333 0.278 1 0.182 0.165 1

6 rs42722878 0.364 0.298 1 0.091 0.087 1 0.545 0.463 1 0.091 0.087 1 0.818 0.483 0.113 0.500 0.375 1 0.273 0.483 0.316 0.333 0.278 1 0.091 0.087 1

7 rs42722887 0.364 0.397 1 0.182 0.165 1 0.545 0.463 1 0.091 0.087 1 0.818 0.483 0.113 0.500 0.375 1 0.273 0.483 0.316 0.333 0.278 1 0.091 0.087 1

8 rs42722900 0.091 0.087 1 0.091 0.087 1 0.091 0.236 0.286 0 0 1 0.636 0.483 0.797 0.500 0.375 1 0.182 0.397 0.217 0.167 0.153 1 0.091 0.087 1

9 rs42722901 0.273 0.351 0.877 0.182 0.165 1 0.5 0.455 1 0.091 0.087 1 0.818 0.483 0.113 0.500 0.375 1 0.273 0.483 0.316 0.333 0.278 1 0.091 0.087 1

10 rs42306198 0 0 1 0.091 0.087 1 0.182 0.165 1 0.091 0.087 1 0.364 0.298 1 0 0 1 0 0 1 0.167 0.153 1 0.091 0.087 1

11 rs17870317 0.636 0.434 0.495 0.636 0.434 0.495 0.545 0.496 1 0.727 0.496 0.427 0.273 0.483 0.316 0.667 0.500 1 0.636 0.434 0.495 0.333 0.444 1 0.455 0.434 1

12 rs17870361 0.455 0.351 1 0.273 0.236 1 0.091 0.087 1 0.364 0.397 1 0.273 0.236 1 0.333 0.278 1 0.455 0.434 1 0 0 1 0.091 0.087 1

13 rs43626955 0.364 0.463 0.811 0.455 0.483 1 0.455 0.351 1 0.273 0.236 1 0.364 0.298 1 0.167 0.153 1 0.545 0.496 1 0.333 0.278 1 0.455 0.434 1

14 rs43626956 0.364 0.463 0.811 0.455 0.483 1 0.455 0.351 1 0.273 0.236 1 0.364 0.298 1 0.167 0.153 1 0.545 0.496 1 0.500 0.375 1 0.455 0.434 1

15 rs43626957 0.636 0.483 0.793 0.273 0.500 0.278 0.636 0.434 0.495 0.364 0.397 1 0.091 0.087 1 0 0 1 0.545 0.496 1 0.500 0.375 1 0.273 0.351 0.877

16 rs42748012 0.545 0.463 1 0.273 0.500 0.278 0.455 0.434 1 0.455 0.351 1 0.455 0.500 1 0.333 0.444 1 0.273 0.236 1 0.333 0.444 1 0.455 0.434 1

17 rs42738663 0.545 0.463 1 0.273 0.500 0.278 0.455 0.434 1 0.455 0.351 1 0.455 0.500 1 0.333 0.444 1 0.273 0.236 1 0.333 0.444 1 0.455 0.434 1

18 rs42311164 0.545 0.397 0.793 0.545 0.463 1 0.273 0.351 0.877 0.273 0.236 1 0.455 0.434 1 0.333 0.444 1 0.364 0.397 1 0.167 0.486 0.303 0.636 0.500 0.884

19 rs42613762 0.182 0.397 0.217 0.091 0.087 1 0.400 0.420 1 0.455 0.351 1 0.273 0.434 0.464 0.167 0.153 1 0.545 0.496 1 0.500 0.486 1 0.273 0.236 1

20 rs41255356 0.545 0.463 1 0.455 0.434 1 0.364 0.390 1 0.273 0.351 0.877 0.545 0.496 1 0.167 0.153 1 0.545 0.397 0.793 0.333 0.278 1 0 0 1

21 rs41774805 0.545 0.397 0.793 0.545 0.496 1 0.364 0.397 1 0.182 0.397 0.217 0.727 0.463 0.264 0 0.444 0.061 0.545 0.463 1 0.333 0.5 0.822 0.273 0.500 0.278

22 rs41720009 0.636 0.483 0.793 0.636 0.483 0.793 0.727 0.496 0.427 0.545 0.397 0.793 0.455 0.351 1 0.167 0.153 1 0.455 0.351 1 0.500 0.375 1 0.455 0.351 1

23 rs41905209 0.273 0.236 1 0 0 1 0.273 0.236 1 0.455 0.483 1 0.182 0.165 1 0.333 0.278 1 0 0 1 0.167 0.153 1 0 0 1

24 rs42803062 0.545 0.463 1 0.636 0.434 0.495 0.455 0.483 1 0.455 0.351 1 0.636 0.483 0.793 0.167 0.153 1 0.364 0.397 1 0.500 0.486 1 0.364 0.496 0.659

25 rs41969933 0.455 0.351 1 0.636 0.434 0.495 0.273 0.236 1 0.545 0.463 1 0.273 0.351 0.877 0.333 0.444 1 0.364 0.298 1 0.167 0.486 0.303 0.273 0.236 1

26 rs42013154 0.364 0.397 1 0.091 0.087 1 0.273 0.236 1 0.182 0.165 1 0.182 0.463 0.118 0.500 0.375 1 0.455 0.351 1 0 0 1 0.273 0.236 1

27 rs42016156 0.889 0.494 0.105 0.455 0.500 1 0.500 0.455 1 0.636 0.434 0.495 0.182 0.298 0.556 0 0 1 0.273 0.236 1 0.500 0.486 1 0.273 0.434 0.464

28 rs42015934 0.455 0.351 1 0.091 0.087 1 0.273 0.236 1 0.091 0.087 1 0.091 0.087 1 0.333 0.278 1 0.364 0.298 1 0.333 0.444 1 0.182 0.165 1

29 rs42451508 0.273 0.236 1 0 0.165 0.095 0.364 0.397 1 0.091 0.087 1 0.636 0.434 0.495 0.333 0.278 1 0.455 0.483 1 0.667 0.444 0.970 0.182 0.397 0.217

30 rs42174698 0.364 0.463 0.811 0.455 0.500 1 0.182 0.165 1 0 0.496 0.001 0 0 1 0.200 0.500 0.476 0.091 0.087 1 0 0 1 0.273 0.483 0.316

31 rs17871172 0 0 1 0.091 0.087 1 0 0 1 0 0 1 0.091 0.087 1 0 0 1 0 0 1 0.167 0.153 1 0 0 1

32 rs42188070 0.273 0.236 1 0.182 0.165 1 0.364 0.496 0.659 0.273 0.236 1 0.182 0.298 0.556 0.167 0.375 0.545 0.364 0.298 1 0.500 0.375 1 0.364 0.397 1

All 0.386 +/- 0.198 0.284 +/- 0.215 0.368 +/- 0.175 0.287 +/- 0.199 0.386 +/- 0.230 0.272 +/- 0.178 0.355 +/- 0.194 0.333 +/- 0.170 0.259 +/- 0.176

*H*_o_ observed heterozygosity

*H*_e_ expected heterozygosity

HWE, *P*-value for the Hardy-Weinberg equilibrium test

AUB, Aubrac, BLA, Blonde d’Aquitaine, CHA, Charolais, HOL, Holstein, LIM, Limousin, MAN, Maine Anjou, MON, Montbéliarde, NOR, Normande, SAL, Salers
