# Supplementary material for: Gene-based single nucleotide polymorphism discovery in bovine muscle using next-generation transcriptomic sequencing
Source: BMC Genomics. 2013 May 7;14:307. doi: 10.1186/1471-2164-14-307 (PMC3751807; doi:10.1186/1471-2164-14-307)
Supplement: Additional file 8: Table S8 — Genetic differentiation (FST) between pairs of cattle populations (above the diagonal) and Reynold’s genetic distance (DR) between pairs of cattle populations (below diagonal) as observed in this study. [file 1471-2164-14-307-S8.docx]

**Table S8**

AUB BLA CHA HOL LIM MAN MON NOR SAL

AUB 0.0000 0.0011 0.0184 0.0862 0.0817 0.0823 0.0529 0.0391 0.0419

BLA 0.0011 0.0000 0.0492 0.1624 0.1248 0.1319 0.1057 0.0655 0.0483

CHA 0.0186 0.0504 0.0000 0.1535 0.1216 0.1276 0.0898 0.0856 0.1057

HOL 0.0901 0.1778 0.1666 0.0000 0.2736 0.2638 0.2059 0.1774 0.2053

LIM 0.0852 0.1333 0.1297 0.3196 0.0000 0.0712 0.0205 0.1001 0.1394

MAN 0.0859 0.1415 0.1366 0.3063 0.0738 0.0000 0.1045 0.1277 0.0802

MON 0.0543 0.1117 0.0941 0.2306 0.0207 0.1104 0.0000 0.0736 0.1109

NOR 0.0399 0.0678 0.0895 0.1952 0.1055 0.1366 0.0765 0.0000 0.1486

SAL 0.0428 0.0496 0.1117 0.2297 0.1501 0.0836 0.1175 0.1609 0.0000

AUB, Aubrac, BLA, Blonde d’Aquitaine, CHA, Charolais, HOL, Holstein, LIM, Limousin, MAN, Maine Anjou, MON, Montbéliarde, NOR, Normande, SAL, Salers
