# Supplementary material for: Gene-based single nucleotide polymorphism discovery in bovine muscle using next-generation transcriptomic sequencing
Source: BMC Genomics. 2013 May 7;14:307. doi: 10.1186/1471-2164-14-307 (PMC3751807; doi:10.1186/1471-2164-14-307)
Supplement: Additional file 9: Figure S1 — Principal Component Analysis. Per cent value in each axis indicates contribution to the total genetic variation. [file 1471-2164-14-307-S9.docx]

**Figure S1**

**
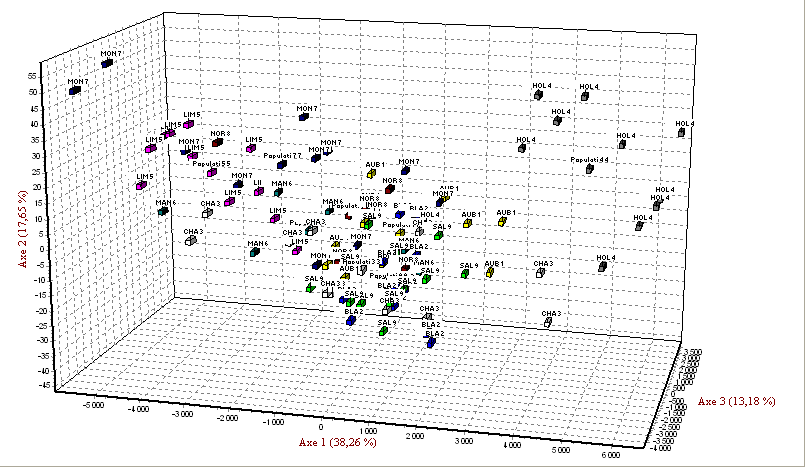
**

AUB, Aubrac, BLA, Blonde d’Aquitaine, CHA, Charolais, HOL, Holstein, LIM, Limousin, MAN, Maine Anjou, MON, Montbéliarde, NOR, Normande, SAL, Salers
